# Accessing a Hidden Conformation of the Maltose Binding Protein Using Accelerated Molecular Dynamics

**DOI:** 10.1371/journal.pcbi.1002034

**Published:** 2011-04-21

**Authors:** Denis Bucher, Barry J. Grant, Phineus R. Markwick, J. Andrew McCammon

**Affiliations:** 1 Department of Chemistry and Biochemistry and Center for Theoretical Biological Physics, University of California at San Diego, La Jolla, California, United States of America; 2 Howard Hughes Medical Institute, University of California at San Diego, La Jolla, California, United States of America; 3 Department of Pharmacology, University of California at San Diego, La Jolla, California, United States of America; National Cancer Institute, United States of America and Tel Aviv University, Israel

## Abstract

Periplasmic binding proteins (PBPs) are a large family of molecular transporters that play a key role in nutrient uptake and chemotaxis in Gram-negative bacteria. All PBPs have characteristic two-domain architecture with a central interdomain ligand-binding cleft. Upon binding to their respective ligands, PBPs undergo a large conformational change that effectively closes the binding cleft. This conformational change is traditionally viewed as a ligand induced-fit process; however, the intrinsic dynamics of the protein may also be crucial for ligand recognition. Recent NMR paramagnetic relaxation enhancement (PRE) experiments have shown that the maltose binding protein (MBP) - a prototypical member of the PBP superfamily - exists in a rapidly exchanging (ns to µs regime) mixture comprising an open state (approx 95%), and a minor partially closed state (approx 5%). Here we describe accelerated MD simulations that provide a detailed picture of the transition between the open and partially closed states, and confirm the existence of a dynamical equilibrium between these two states in apo MBP. We find that a flexible part of the protein called the balancing interface motif (residues 175–184) is displaced during the transformation. Continuum electrostatic calculations indicate that the repacking of non-polar residues near the hinge region plays an important role in driving the conformational change. Oscillations between open and partially closed states create variations in the shape and size of the binding site. The study provides a detailed description of the conformational space available to ligand-free MBP, and has implications for understanding ligand recognition and allostery in related proteins.

## Author Summary

Maltose-binding protein (MBP) is a bacterial protein involved in nutrient uptake. Crystallographic studies have revealed two stable conformations: a ligand-free open form and a liganded closed form. The interconversion between the 2 forms has been traditionally viewed as a ligand “induced” process. However, recent NMR experiments have suggested that the ligand-free protein is flexible enough to visit partially closed states. Because these states may display higher affinity for the ligand than the open state, ligand binding may proceed via a “conformational selection” of the most geometrically and chemically favored conformer. Mechanistic details of this process remain elusive as atomic structures of relevant intermediate conformations are currently lacking. In this study, we use atomistic simulations to characterize the flexibility of the MBP protein, and confirm the existence of a hidden semi-closed state. The relative stabilities of the two states is predicted and corroborated with existing experimental data. A key finding of the study is that the closed form of the protein – adopted by the ligand-bound form – is not observed in the simulations of the ligand-free protein. This has implications for understanding ligand binding mechanisms in MBP and related proteins.

## Introduction

Periplasmic Binding Proteins (PBPs) are major components of the bacterial cell envelope that are involved in nutrient uptake and chemotaxis [1], [2]. Gram-negative bacteria use PBPs to transport ligands into the cytosol by association with a membrane-bound ATP-binding cassette (ABC) transporter [3]. Gram-positive bacteria differ in that they employ a slightly different design, in which the PBPs motif is directly attached to a membrane-anchored receptor. In addition, several mammalian receptors contain extracellular ligand binding domains that are homologous to PBPs. These include glutamate/glycine-gated ion channels such as the NMDA receptor; G protein-coupled receptors, including metabotropic glutamate, GABA-B, calcium sensing, and pheromone receptors; and atrial natriuretic peptide-guanylate cyclase receptors. Many of these receptors are promising drug targets [4]. The structures of PBPs (∼100 X-ray structures) have been called a ‘gold mine’ for studying the general mechanisms of protein-ligand recognition [2], as PBPs have been identified that can transport a large variety of substrates, including: carbohydrates, amino acids, vitamins, peptides, or metal ions [5]. The affinity of PBPs for diverse substrates also make them ideal templates for the design of diverse *in vitro* and *in vivo* biosensors with tailored properties [6].

**Figure 1:**
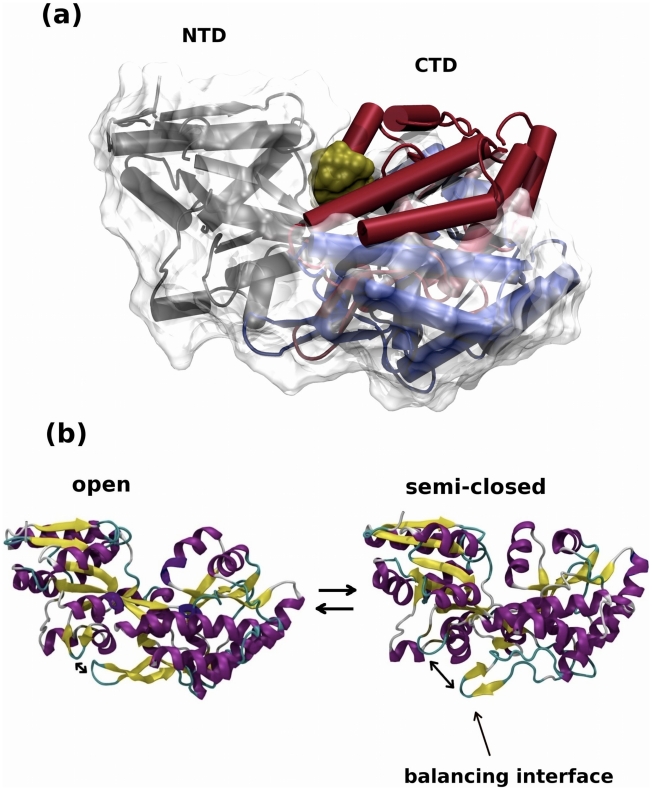
Maltose Binding Protein. (a) Cartoon representations of the open and closed X-ray structures of MBP (pdb code 1omp [31] and 3mbp [54]). The NTDs of apo and holo MBP are superimposed (grey), and the displacement of the CTD in the open (blue) and closed (red) conformations is shown. The ligand maltotriose is shown in yellow (b) The NMR experiments of Tang et al [12] indicate that the ligand-free protein is in a preexisting dynamical equilibrium between an open and a partially closed conformation.

Maltose binding protein (MBP) is a part of the maltose/maltodextrin system of *Escherichia coli*, which is responsible for the uptake and efficient catabolism of maltodextrins. MBP is the prototypical member of the PBP superfamily. It has been the subject of extensive study due to its importance in various biological pathways [2], [6], and its utility as an affinity tag for protein expression and purification [7]. The protein folds into two domains of roughly equal size: the C terminal domain (CTD), and the N terminal domain (NTD) (Fig. 1). The two domains are connected via a short helix and a two-stranded β-sheet that form an interdomain hinge region. Like other PBPs, the binding site of MBP is located on the interdomain cleft between domains. X-ray structures of MBP solved in the presence and absence of ligand indicate that the protein undergoes an important conformational change from an open to a closed state in the presence of the ligand, the effect of which is to better stabilize the ligand by reducing the size of the cleft [2]. The conformational change has been dubbed the “Venus Fly-Trap Mechanism” [8] due to its resemblance to the traps on the carnivorous plant that closes only when stimulated by prey.

An ‘induced-fit’ mechanism [9] is often invoked to describe the ligand recognition process. In this scenario, the ligand participates in remodeling the binding site by interacting directly with the protein. Alternatively, it is also possible that the apo protein already exists in a mixture of open and closed conformations. In which case, the ligand would play a more passive role shifting the equilibrium toward the closed state, a mechanism traditionally described as ‘conformational selection’, or ‘population shift’ [10]. Computer simulations and NMR studies are often needed to distinguish between the two scenarios, as X-ray structures typically do not provide detailed information about the *ensemble* of conformations available to the ligand-free protein [11].

Until recently, the bulk of our understanding of the mechanism of substrate recognition in MBP came from crystallographic studies that indicated only two possible conformations, a ligand-free open, and a ligand bound closed structure. In 2007, Tang et al. [12] reported the first NMR paramagnetic relaxation enhancement (PRE) measurements on apo MBP. By attaching a spin label (TEMPO) on the NTD and CTD of the apo protein, domain hinge-bending motions could be studied. These measurements indicated the existence of a dynamic equilibrium between a major open state, and a minor partially closed state. Because experimental PRE rates for MBP could not be explained either by the X-ray crystal structure of the apo-state (open conformation), nor by the ligand-bound closed-state, it was possible to postulate that a partially closed structure exists. The transition between an open, and a partially closed state, was determined to involve a rotation around the hinge region. The best agreement between computed and experimental PRE and Residual Dipolar Coupling (RDC) data, was obtained by considering that substrate-free MBP exists in equilibrium between a major, open state (95%) and a minor, semi-closed state, populated ∼ 5% of the time, which corresponds to a very small energy difference between the two states (∼2 kcal/mol). The time-scale of the exchange between states was estimated to be between 20 ns to 20 µs [12].

From a theoretical point of view, it is understood that a pre-existing equilibrium between different PBP conformations could play an important role in facilitating ligand recognition [13]. However, it remains a considerable challenge to access, using fully atomistic MD simulations, a detailed statistical analysis of slow conformational dynamics in proteins mediated by such hinge-bending motions. In the past decade, the development of increasingly efficient simulation algorithms has led to a large number of theoretical studies using Molecular Dynamics (MD) simulations to probe the intrinsic dynamics of PBPs [14], [15], [16], [17], [18], [19], [20], [21], [22]. In 2003, Pang et al. [14] studied the glutamine binding protein (GlnBP) using ∼5 ns MD simulations. They observed large vibrations in the apo protein in the direction of a closed structure, and found that the open apo structure was more flexible than the closed structure. Subsequently, Pang et al. [16] confirmed that this is a general result by performing a comparative study of different PBPs, which also showed that different PBPs could display slightly different dynamical properties. The authors also observed that the opening and closure rate in the presence of a substrate could be fast (nanosecond time-scale), even though they also noted that obtaining converged sampling for the opening and closure events was challenging on the nanosecond time-scale. In 2006, Kandt et al. [17] performed longer MD simulations on BtuF, a protein involved in vitamin B_12_ uptake. Using 12 simulations of 30–50 ns each, they were able to observe the initiation of opening and closing motions in both apo and holo simulations, with larger motions in the apo simulations. This behavior of the protein was interpreted to be compatible to the Venus Fly-trap model. The observation of enhanced molecular flexibility in the open state was confirmed by other groups for similar PBPs, such as the iron binding proteins FhuD [18], and FitE [19], and the heme binding proteins, ShuT, and PhuT [20]. In 2009, Loeffler and Kitao [21] studied GlnBP in the open liganded form, and reported closing events occurring during the simulations. Taken together, these MD studies on PBPs have helped characterize the intrinsic flexibility of ligand-free PBPs on the nanosecond time-scale. The consensus of opinion from these calculations is that the ligand recognition proceeds through a Venus flytrap mechanism, and that the apo PBPs structure is very flexible, with a tendency to oscillate along the modes that lead from the open to the closed structure.

In 2005, a simulation study of the MBP protein was carried out by Stockner et al. [22]. Using 4 MD simulations of 30 ns, started from both open and closed states, with and without substrate, the authors could show that the ligand-free MBP structure (open form) naturally evolves toward a closed state in the presence of a substrate. Similarly, the closed state was found to evolve toward an open state when the substrate was removed. The rapid time-scale of this conformational change was consistent with experimental rate constant for sugar binding ∼1–2×10^7^ M^−1^ s^−1^ [23], which suggests a rate of closure around 30–50 ns. However, the time-scale of the simulations was too short to observe any pre-existing equilibrium in apo MBP between an open and a partially closed conformer. This can be explained by the presumed slow exchange rate (20 ns to 20 µs)[12] between the two conformations of apo MBP.

In this paper, we have used accelerated Molecular Dynamics (aMD) simulations [24] of MBP to show that the apo protein exists in a dynamical equilibrium between an open and a semi-closed conformation. A number of methods have been developed to enhance the sampling of slow conformational changes in proteins, including targeted MD [25], and conformational flooding [26]. However, within the framework of this study, a key advantage of aMD is that it allows us to study the conformational behavior and dynamics of the protein without using a pre-defined reaction coordinate. In previous studies, aMD has been successfully employed to study slow time-scale dynamics in proteins, such as HIV-protease [27], ubiquitin [28], IKBA [29] and H-Ras [30]. The enhanced conformational space sampled by aMD has also been shown to significantly improve the theoretical prediction of experimental NMR observables, such as residual dipolar couplings, scalar J-couplings [28] and chemical shifts [29], which are sensitive to dynamic averaging on the micro- to millisecond time-scale. In this paper, we show that aMD simulations successfully allow the study of the transition from the open state of apo MBP to the hidden semi-closed conformation. This provides the first atomistic view of the transition between open and partially closed states in a PBP. NMR parameters computed from the simulations agree well with experiments. Free energy calculations, and continuum electrostatics calculations are used to provide new insights into the mechanism and energetics of the exchange between the open and semi-closed states of apo MBP.

## Methods

The simulation system was constructed using the crystal structure of ligand-free MBP in the open state (pdb code: 1omp) [31]. The MBP structure was solvated in a box of 13,410 TIP3P water molecules [32]. 8 Na^+^ ions were used to neutralized the system, and an ionic strength of 0.15 M was created by adding 34 Na^+^ and Cl^-^ ions. The water and ions were first equilibrated. Then after 1,000 steps of minimization, the system was equilibrated in the presence of Cartesian restraints, slowly reducing the restraining force on all protein atoms (20, 10, 5, 0 kcal/mol Å^2^) in four steps of 20 ps each. The MD simulations were carried out with the AMBER 10 package [33] with the ff03 force field [34] and constant pressure periodic boundary conditions. A time step of 2.0 fs was used, together with the SHAKE algorithm [35] to constrain the bonds connecting hydrogen atoms. The temperature was regulated using a Langevin thermostat [36] with a coupling constant of 2.0 ps^−1^. The pressure was maintained at 1 bar using the Berendsen weak-coupling algorithm [37] with a coupling constant of 2.0 ps. Electrostatic interactions were evaluated using the smooth Particle Mesh Ewald method [38], with a real space cutoff of 8.0 Å. The nonbonded interactions were truncated at 12.0 Å, and the neighbor list was updated every 3 steps. Dual boost aMD simulations [39] were performed. Prior to the aMD simulations, a conventional MD simulation was performed for 2 ns to obtain the average total potential energy (V_0, tot_  =  −243,540 kcal/mol), and the average torsional potential energy (V_0, tors_  = 3900 kcal/mol). The average bias potentials (ΔV_tors_, and ΔV_tot_) were chosen to be both roughly equal to ∼ 40 kcal/mol. To do so, we used the following acceleration parameters: [E_b, tot_ – V_0, tot_, α]  =  [10,000 kcal/mol, 10,000 kcal/mol], for the total boost, and: [E_b, tors_ – V_0, tors_, α]  =  [1300 kcal/mol, 260 kcal/mol], for the torsional acceleration. In order to limit possible artifacts arising from a particular choice of parameters or force field, the simulations were repeated with the CHARMM27 force field [40] and the NAMD2 code [41] for comparison. Simulations were carried out in the NVT ensemble, using a time step of 2.0 fs in combination with the SHAKE algorithm. The temperature was regulated with a Langevin thermostat, using a damping coefficient of 5 ps^−1^. AMD simulations were performed using only a single boost on torsional angles, with [E_b, tors_ – V_0, tors_, α]  =  [2400 kcal/mol, 1000 kcal/mol]. The average boost potential (ΔV_tors_) in these simulations was also around 40 kcal/mol. To accelerate further the exploration of the conformational space, multi-copy simulations were performed [42]. Six different types of simulations were used, as detailed in Table 1. Each set of simulations was repeated 5 times, bringing the total simulation time to ∼1 µs. In order to study the enhanced conformational space sampling observed in the aMD simulation, a principal component analysis was performed, and the interdomain closure angle (θ) was computed during the trajectories. The interdomain angle was defined as the angle between the centers of mass of the NTD, the hinge region, and CTD (see Stockner et al. for details [22]).

**Table 1:** Type of simulations performed: 50 ns MD and aMD simulations (aMD  =  accelerated MD).

Name	Method	Initial Conformation	IntermediateConformation	Final Conformation	θ_ min_-θ_ max_
Sim1	MD (AMBER)	Open	Open	Open	155–161
Sim2	aMD (AMBER)	Open	Semi-Closed	Open	138–159
Sim3	MD (CHARMM)	Open	Open	Open	154–160
Sim4	aMD (CHARMM)	Open	Semi-Closed	Open	136–160
Sim5	MD (AMBER)	Semi-Closed	Semi-Closed	Semi-Closed	145–149
Sim6	MD (CHARMM)	Semi-Closed	Semi-Closed	Semi-Closed	146–150

The simulations were used to study the conformational space accessible to apo MBP. The range of interdomain angles explored during the simulations is given for comparison with X-ray structures of MBP in the open (θ = 161°), and the closed (θ = 137°) conformations.

The Adaptively Biased Force (ABF) method [43], [44] was used to compute the free energy required to displace the balancing interface (residues 175–184). ABF simulations were performed for 50 ns and repeated 5 times starting from both the open and semi-closed conformer to collect statistics. The distance between residues Asn100 and Lys175 was used as the reaction coordinate to pull the balancing interface away from the NTD. The reaction coordinate was scanned between 4 and 15 Å, every 0.1 Å, using 500 samples in a bin prior to application of the ABF protocol.

Continuum electrostatic calculations were performed using the MM-PBSA (Molecular Mechanics/Poisson-Boltzmann Surface Area), and the MM-GBSA (Molecular Mechanics/Generalized Born Surface Area) methods [45]. These simulations were used to highlight the different energy terms that contribute to the stability of the protein. To obtain converged sampling, within a statistical precision of ∼ 0.5 kcal/mol, 40 MD simulations of 1 ns were started from different sets of initial coordinates in the open, and semi-closed conformations. Snapshots were saved every 5 ps, and processed to analyze the different energy terms, leading to a total of 8000 MM-PBSA calculations. The relative free energy between the two conformational states of apo MBP was found to be in excellent agreement with the NMR experiments of Tang et al. (within 1 kcal/mol). It suggests that the associated MM-PBSA errors in this study are reasonable, and considerably smaller than the errors sometimes reported in the calculations of protein-ligand interactions (>10 kcal/mol) with the MM-PBSA method [46].

### Calculation of transverse Paramagnetic Relaxation Enhancement (PRE) rates

In the framework of the Solomon-Bloembergen Model-Free formalism (SBMF) [47], the transverse paramagnetic relaxation enhancement rate, Γ_2_, between the electron located at the paramagnetic center associated with the spin-label and a ^1^H nucleus is given by:
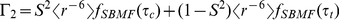
(1)where *f*_SBMF_ is defined as:
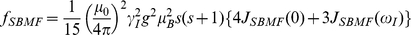
(2)


In the above equations, *r* is the distance between the electron and ^1^H nucleus, *s* is the electron spin quantum number, *g* is the electron g-factor, γ_I_ is the proton gyromagnetic ratio, μ_0_ is the permeability of a vacuum, μ_B_ is the magnetic moment of the free electron, *S^2^* is the order parameter associated with the interaction vector between the electron and the ^1^H nucleus and ω_I_/2π is the Lamor frequency of the proton. The reduced generalized spectral density function, *J_SBMF_*(ω) is given by:
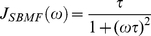
(3)


The associated correlation time, τ, is either τ_c_ or τ_t_, which are defined as:
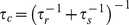
(4)and
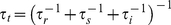
(5)where τ_r_ is the rotation correlation time of the molecule, and τ_i_ is the correlation time for internal motion of the interaction vector between the electron and the ^1^H nucleus assuming that the internal motion is not coupled to the overall tumbling of the molecule. τ_s_ is the effective electron relaxation time, which in the present work is ignored as it is assumed to be substantially longer than the rotation diffusion time of the molecule and so has a negligible effect on the associated spectral density function [48]. The reader is referred to Ref. 47 for complete derivations and discussion of the above equations.

The accelerated molecular dynamics simulations identified two states for apo-MBP (see Results): A major open state and a minor semi-closed state. For each state, six structures were obtained from the aMD simulations and in each case, the Asp41 residue was modified to a non-standard CYS-TEMPO spin-label residue. The force-field for this non-standard residue was generated using the AMBER force-field (GAFF) [49]. A simulated annealing protocol was performed with Cartesian restraints applied to all solute atoms other than those associated with the modified CYS-TEMPO residue, in order to generate different configurations for the CYS-TEMPO group. Four different CYS-TEMPO configurations were generated for each of the six initial starting structures in each state. In this way, 48 systems were generated (24 systems in each state). Each system was placed at the center of a pre-solvated water box and brought to equilibrium at 300K, 1-bar pressure. A 20-ns production MD run was performed for each system and the average of the inverse distances to the power six, <r^−6^>, the associated order parameters, S^2^, and internal correlation times, τ_i_, for the interaction vector between the electron located at the nitroxide group of the TEMPO spin-label and all amino-^1^H nuclei were calculated. Transverse paramagnetic relaxation rates for each state (open and semi-closed) were calculated from each simulation using equation (1) and averaged over the 24 simulations. Assuming the relative population of the open and semi-closed states to be 95%-5% (see MM/PBSA analysis) the final theoretical free-energy weighted combined open/semi-closed PRE data was obtained.

## Results

### Accelerated simulations of apo MBP

**Figure 2:**
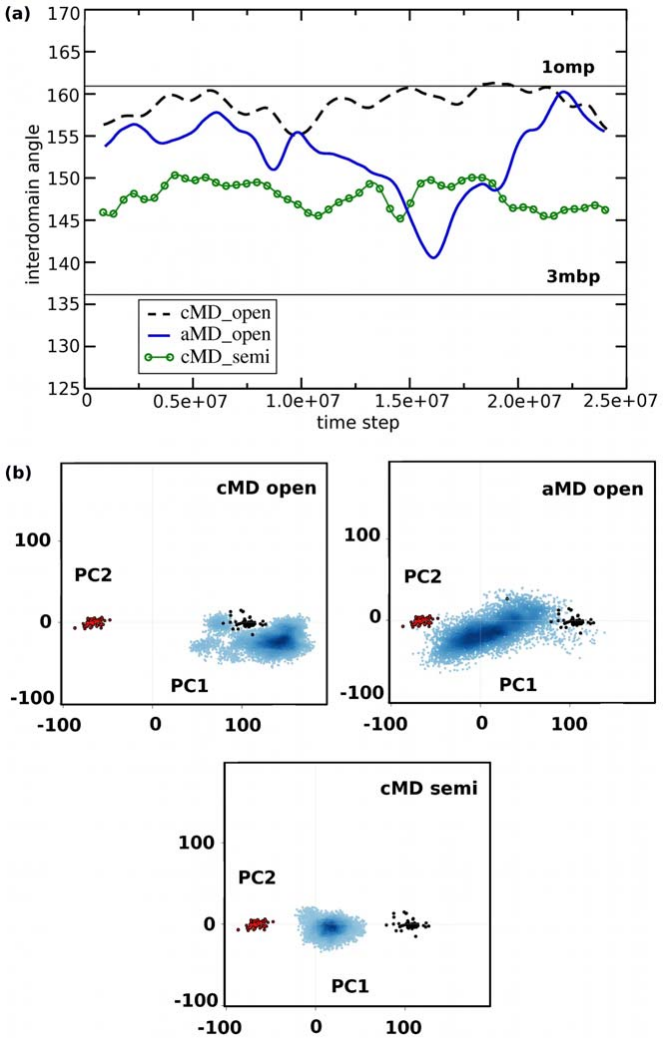
Accelerated MD trajectories. (a) Interdomain angles shown here for one set of AMBER simulations (sim1, sim2, sim5). The horizontal lines correspond to the interdomain angle in X-ray structures for the open (apo), and closed (holo) forms. (b) Principal Component Analysis (PCA): The first two PCs were calculated using available X-ray structures in the open and closed states (black, and red points, respectively). The trajectories (in blue) are projected on the space defined by the first 2 principal components (PCs).

Conventional and accelerated MD simulations were performed to study the conformational behavior and dynamics of MBP. When started in the open state (Sim 1 and Sim 3), conventional simulations (50 ns) were unable to explore a new conformation, as shown by the limited range of interdomain angles observed during theses simulations (Table 1). The MBP structure remained open throughout the simulation at an interdomain angle, θ constant between 154 and 161° (Fig. 2(a)).

In comparison, accelerated MD simulations, initiated from the open state (Sim2, and Sim4), transition to a semi-closed state, indicated by a change of ∼ 3 Å in the Cα RMSD, followed by a sharp decrease in θ from ∼ 160° to ∼ 145°. These simulations were repeated 5 times, and in all cases were shown to visit a partially closed state and to eventually come back to the open state. The interdomain angle during a typical aMD trajectory is drawn in Fig. 2(a), as a function of the number of aMD steps. The number of aMD steps is used in this plot, since the ‘real’ time-scale in aMD is non-linear, and difficult to assess accurately. However, previous work on proteins [27], [28], [29], [30] has suggested that the level of acceleration used in this study corresponds to sampling the protein motion on the micro- to millisecond time-scale. NAMD2 simulations with CHARMM force field were performed for comparison, and an identical transition to a semi-closed structure was observed.

Standard MD simulations were then started from the semi-closed conformation, to show that this new conformational state is indeed stable. These additional simulations displayed a stable interdomain angle (at average values of θ = 146(3)°, and 148(3)°, for sim5, and sim6, respectively). Interestingly, these θ values are exactly intermediates between the interdomain angles of the X-ray structures in the fully open (θ = 161°) and fully closed (θ = 137°) conformations. Moreover, the structures obtained with CHARMM and AMBER were almost identical when superimposed, with a backbone RMSD <1.5 Å. This should be compared to the larger RMS deviations observed with respect to the open (3.0–3.5 Å), and closed forms (2.0–2.5 Å).

To further characterize the conformational space sampled in the simulations, and show that the identified semi-closed state occupies a local energy minimum on the PES, the trajectories were analyzed in terms of their principal components of atomic fluctuations [50]. All publicly available X-ray structures of MBPs were used to perform a principal components (PC) analysis (see Ref. [51] for details) supplementary material for details). The first two PCs were found to capture >95% of the variance associated with the open and closed structures, and were therefore used to further analyze the trajectories. As can be seen in Fig. 2(b), the X-ray structures display two well-defined clusters on the PC-space that correspond to the open (ligand-free) and closed (with bound ligand) conformers. A representative MD simulation initiated in the open state (sim1) is shown to explore only the local vicinity of the open X-ray structures, whereas a representative aMD simulation (sim2) is able to explore a region of the PC-space that is intermediate between the open and closed form. In addition, when starting a MD simulation from the semi-closed conformer (sim5), the simulation remains in the basin corresponding to the semi-closed conformer, which indicates that a stable minimum on the potential energy surface exists for this structure, between the fully open and fully closed states.

### Mechanism and energetics of the transformation

After establishing the existence of a hidden semi-closed state of MBP, the trajectories were further analyzed to gain new insight into the pre-existing equilibrium dynamics between the open and semi-closed states. Visual inspection of the trajectories reveals that a flexible part of the protein, called the balancing interface (Fig. 1), is displaced during the transformation. The balancing interface is a beta-hairpin motif that belongs to the CTD, and which interacts only through weak vdW interactions with the NTD. Prior to the conformational change, the contact between the tip of the balancing interface and the NTD is lost. This event is generally followed by a rotation of the two globular domains of ∼ 15° around the hinge β-sheet residues.

**Figure 3:**
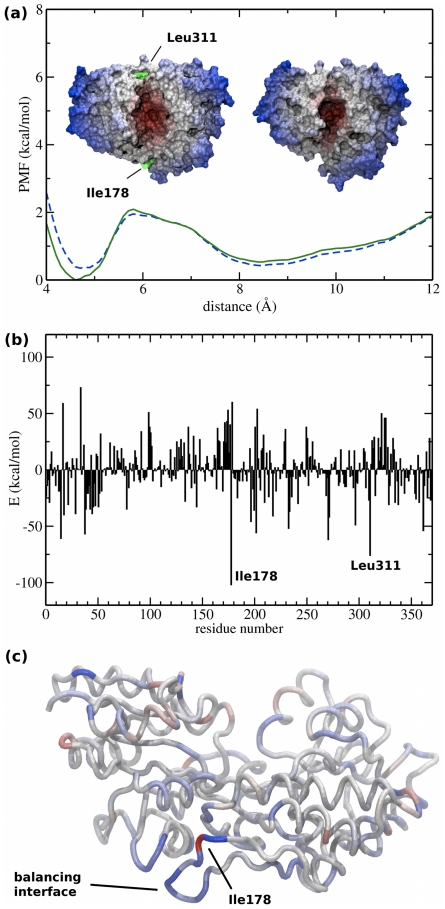
Open and semi-closed conformations. (a) a view from the top of the protein shows the different size and shape of the ligand binding site in the two conformations. A red color indicates that the atoms are close to the binding site, and a blue color that they are >20 Å away. The forward and reverse potential of mean force (PMF) for the displacement of the balancing interface is shown. Residues Ile178 and Leu311 (green) are located in the hinge region, and are buried inside the protein in the semi-closed state. (b) Per residue decomposition of the (MM/GBSA) free energy showing the contribution of each residue to the stability of the open versus the semi-closed state. (c) Color representation of the energy contribution to the open state (blue), versus the semi-closed state (red).

To establish the role of the balancing interface in the conformational change, the ABF method was used to introduce an external force that artificially pulls the balancing interface away from the NTD. 5 ABF simulations were carried out for 50 ns, starting from the open conformer. In each of these simulations, once the balancing interface was no longer interacting with the CTD, a spontaneous conformational change occurred leading to the semi-closed state. In addition, 5 ABF simulations were started from the semi-closed conformer. In these simulations, as the distance between the tip of the balancing interface and the NTD was decreased, the structures reverted back into the open conformer, showing that the process is reversible. These simulations also provide information about the free energy barrier for the displacement of the balancing interface (∼2 kcal/mol) (Fig. 3(a)). The involvement of the balancing interface in the transition to the semi-closed state is consistent with two mutational studies that have previously suggested that it plays a role in the conformational change of MBP [52], [53], and described the balancing interface as a “spring” that maintains the protein open. Moreover, X-ray crystal structures of both the apo (open) and holo (closed) states of MBP show that the contact between the NTD and the balancing interface is lost only in the closed state [54], [55], [56]. Our simulations are consistent with this representation, and indicate that the balancing interface acts as a “molecular switch” that initiates the conformational change when displaced.

To provide insight into the energetics of the transformation, we have analyzed the relative free energy changes for each residue as a function of the conformational state. This was done by performing an MM/GBSA per residue free energy decomposition analysis for both the open and semi-closed states. The results, presented in Fig. 3, indicated as expected that residues near the tip of the balancing interface help stabilize the open conformation. In contrast, several non-polar residues near the hinge region were found to help stabilize the semi-closed conformation, by re-packing and dramatically reducing their solvent-accessibility during the conformational change. In particular, residues Ile178 and Leu311 were found to be solvent-exposed in the open state, but buried inside the protein in the semi-closed state (Fig. 3(a)). Ile178 was able to interact with another non-polar residue, Ile333, in the semi-closed state. The composition of the balancing interface is interesting in this respect, because it contains several hydrophobic residues that are all located on one side of the beta hairpin motif (Phe169, Ile178, Val181, Val183), while the other side contains side-chains that are hydrophilic (Lys170, Glu172, Lys175, Asp177, Lys179, Asp180, Asp184). Visual inspection of the trajectories suggests that the opening of the flap momentarily increases the solvent accessibility of non-polar residues in the hinge region (Ile178, Val181, Ile329, Ile333). This most likely destabilizes the open structure and helps drive the conformational transition to the semi-closed state. The proposed mechanism is consistent with the mutational study of Telmer and Shilton [52], which has previously shown that certain mutations in MBP far away from the sugar binding pocket can increase the affinity for maltose and reduce the off-rate. These mutations (Met321, Gln325, and residues 171–177) were found to occur in residues that are important for protecting non-polar regions of the protein from the solvent, in particular residues Ile178, and Leu311. The picture that emerges is one in which the improved binding affinity for maltose in the mutants could be explained by the fact that these mutants are more likely to find themselves in a semi-closed state to avoid a large exposure of hydrophobic regions of the protein.

To learn more about the different energy terms, the various enthalpic and entropic contributions to the total free energy in the open and semi-closed states of MBP were further analyzed using MM-PBSA calculations. The results are shown in Table 2, (force field terms for the protein, polar and non-polar solvation, and entropy term derived from a normal mode analysis).

**Table 2:** MM-PBSA calculations used to decompose different energy terms for the open and semi-closed conformers: (ELE  =  electrostatic, VDW  =  van der Waals, INT  =  torsional energy, and their sum gives the protein energy: PROT  =  ELE + VDW + INT), the solvation terms (PBSUR  =  non-polar contribution, PBCAL  =  polar contribution, and the total contribution from solvation SOLV  =  PBSUR + PBCAL), the entropic contribution (TS), and the total free energy PBTOT  =  PROT + TS + SOLV. (STE  =  statistical error).

*(kcal/mol)*	*1. ELE*	*2. VDW*	*3. INT*	*(1+2+3) 4. PROT*	*5. PBSUR*	*6. PBCAL*	*(5+6)* *7. SOLV*	*8. TS*	*(4+7)* *9. PBTOT*
1. Open	-6853.7	-1611.3	7864.4	***-600.6***	760.8	-4564.9	***-3804.1***	-4151.1	**-4404.7**
2. Semi-closed	-6766.4	-1616.2	7875.6	***-506.7***	768.4	-4664.7	***-3896.3***	-4150.9	**-4403.2**
ΔG_(1 - 2)_	-87.3	4.8	-11.2	***-93.7***	-7.6	99.8	***92.2***	-0.2	**-1.5**
STE	1.9	0.3	0.4	***1.8***	0.2	1.7	***1.6***	1.2	**0.5**

In agreement with the experimental study of Tang et al. [12], the two conformations were found to have roughly the same total energy, and the open structure was found to be slightly more stable (ΔG ∼ −2 kcal/mol). Interestingly, the protein electrostatic energy was found to help stabilize the open conformational state, which as discussed above, is likely to be due to the favorable interaction between the tip of the balancing interface and the NTD. In contrast, solvation effects were found to favor the semi-closed state to a similar degree. This analysis suggests that the open conformation is more stable because the favorable electrostatic interactions in the balancing interface are slightly superior to the destabilizing hydrophobic effect. However, as the balancing interface moves into solution, the repacking of non-polar residues leads to a conformational change into the slightly more compact semi-closed state.

**Figure 4:**
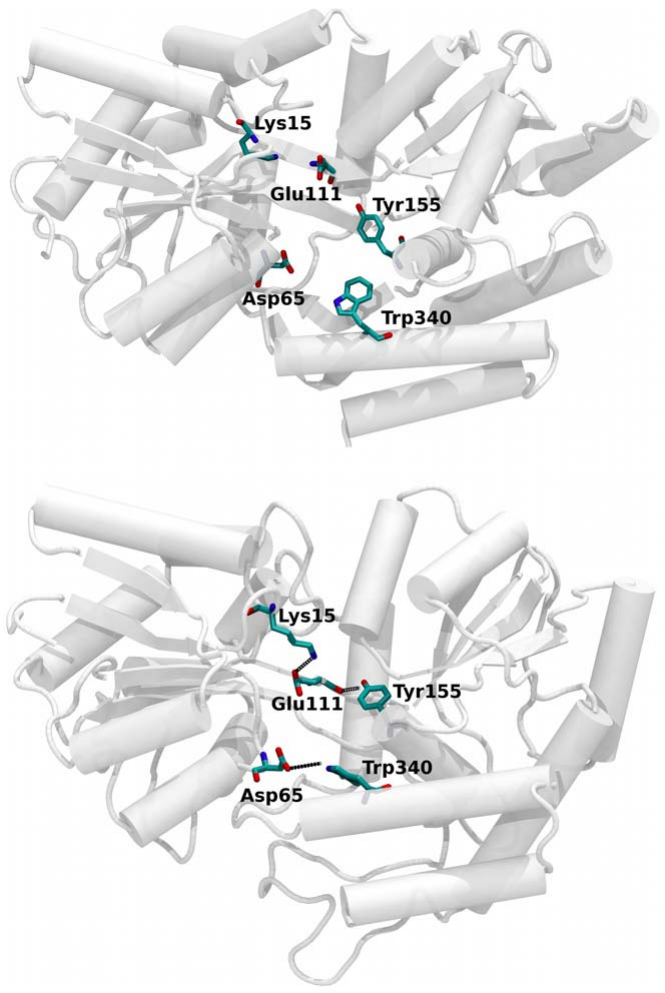
Interdomain hydrogen bonds. Two hydrogen bonds, absent in the open state (top), are formed in the semi-closed state (bottom): Trp340 (CTD) is found to interact with Asp65 (NTD), and the backbone carbonyl of Glu111 interacts with Tyr155. Both Trp340, and Tyr155 residues are sugar-stacking residues that are also involved in ligand recognition.

Interestingly, close-range interactions between the two domains were found to be very limited. In a previous study, Stockner et al. [22] found that, during the transformation into the closed state in the presence of a substrate, a salt-bridge between the Glu111 (hinge region) and Lys15 (NTD) residues can assist the conformational change in holo MBP transition into the closed state. The authors also reported a temporary interaction between Tyr155 (CTD) and the backbone carbonyl of Glu111. Here, we observed a similar interaction between Glu111 and Lys15; however, we also find that the salt-bridge is not always formed in the semi-closed state. The interaction between Tyr155 and Glu111 is also observed and appears to help stabilize the semi-closed conformation (Fig. 4). In addition, a second stabilizing hydrogen bond was discovered between Asp65 (NTD) and Trp340 (CTD). These two interactions, shown in Fig. 4, help stabilize the semi-closed state.

### Comparison with NMR data

**Figure 5:**
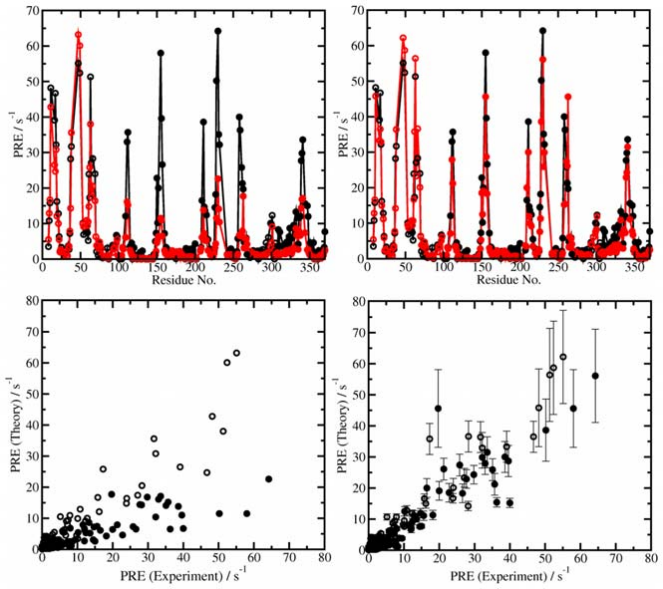
Computed and experimental paramagnetic relaxation enhancement rates. In the upper panels, the black curves are the experimental results, and the red curves are the theoretical results. When only MD trajectories in the open state are considered (left panels), the agreement with experimental PRE is not optimal. However, the agreement is significantly improved by including simulations started in the semi-closed conformation (right panels). The PRE data for the N-terminal domain are represented using open circles, and the PRE data for the C-terminal domain and linker regions are represented using closed circles.

As previously discussed, nuclear magnetic resonance-based PRE data provides very accurate information about the conformational behavior of proteins. Having identified a pre-existing dynamic equilibrium for apo MBP between an open and semi-closed state from the aMD simulations and calculated the relative free energies of these states using an MM/PBSA analysis, the *validity and accuracy* of our theoretical results can be determined by back-calculating the experimental data. To confirm that the simulations can sample accurately the conformational space of MBP, we have computed the PRE rates directly from structures obtained in MD trajectories. In Fig. 5, we compare the PRE calculated for several simulations started from the open conformations, and for the case where the back-calculated data is the product of averaging over all the MD simulations (see PRE Methods section for details). The agreement with experiment was significantly improved in that case (Fig. 5).

In addition to the NMR PRE data, there is a very high degree of structural similarity between the semi-closed state that we obtain from our aMD simulation study and distances reported in the study of Tang et al. For example, the distance between residues Glu153 (CTD domain) and Asp14 (NTD domain) was reported to be ∼ 13.8 Å, in the semi-closed state. Excellent agreement was obtained here in our simulations in the semi-closed state (14.5±0.9 Å). Moreover, this value is quite different from the average distance measured in X-ray structures corresponding to the open, or closed conformations (17.1–19.3 Å, and 9.5–10.2 Å, for open, and closed structures, respectively), which further demonstrates that the semi-closed conformation is a distinct state.

## Discussion

Our computer simulations confirm the results of Tang et al. and solidly establish the existence of a preexisting dynamical exchange in MBP between an open, and a semi-closed conformational state. In order to rapidly exchange between states, the protein must exist in two conformations with similar free energies that are connected by a relatively flat free energy pathway. In principle, this could be achieved by using a variety of residues to assist the transformation, such as, “hook-and-eye residues” that can connect different regions of the protein during an exploration phase, or “anchor-and-latch residues” that may be important in a later stage to lock the protein into a stable conformation.

In MBP, we have found that large hydrophobic residues are important because they can be used to help stabilize the partially closed conformation. This may be a principle used by other hinge-bending proteins to increase compactness, as the solvent accessibility of hydrophobic side-chains is easier to reduce in compact structures [57]. Interestingly, in MBP the open state is slightly more stable than the semi-closed state, which raises the question of how this is achieved. To answer this question, we performed continuum electrostatic calculations, and found that electrostatic interactions between the balancing interface and the NTD help to maintain the open structure. Similarly, the displacement of the balancing interface into the solvent was generally followed by a spontaneous conformational transition into the semi-closed state. Thus, the picture that emerges is one in which the re-packing of non-polar residues >20 Å away from the ligand binding site can affect its properties. This observation is consistent with a large number of mutational studies showing the important structural role played by non-polar residues near the hinge region [52], [53], [58], [59], [60].

From a design perspective, the important role of hydrophobic residues in facilitating the hinge-bending motion could be seen as counter-intuitive, as one could expect short-range electrostatic interactions between the two globular domains to be more effective than solvation effects. For example, electrostatic interactions are known to be very effective in protein-protein recognition [61], [62], enzymatic catalysis [63], ion channel selectivity [64], and protein-ligand interactions [2], [65]. In contrast, the simulations suggest that close-range electrostatic interactions do not contribute significantly to the hinge bending motion, as the associated contacts between the NT and CT domains are very limited. Several intra-domain salt-bridges near the binding site were seen to break and reform during the simulations (such as Glu153 and Arg344, Glu45 and Arg66, Arg367 and Asp363), but the formation of salt-bridges between the two domains did not occur. Interestingly, a similar conclusion was reached by Sinha et al. [66], who analyzed the role of salt-bridges from a database of 36 hinge-bending proteins. They found that close-range electrostatic interactions are largely absent between moving domains. Instead, large non-polar buried surface areas were often found to be used to create a smooth energy landscape where thermal fluctuations can drive hinge-bending motions.

Our simulations of the apo protein indicate that the fully closed state is never reached in the absence of a ligand. This suggests that an induced-fit step may still be required to reach the fully closed state. The existence of a pre-existing equilibrium dynamics in MBP between open and semi-closed conformations is most consistent with a two-step mechanism for ligand-recognition, in which the first step involves the formation of an encounter complex in the semi-closed state via an population shift mechanism, followed by a fast induced fit rearrangement, resulting in the fully closed state. Interestingly, the pre-existing dynamical equilibrium between the open and semi-closed states may not be shared by all PBPs [67]. Studies by Bermejo et al. of GluBP indicate that this system does not seem to achieve a ligand-free open to partially closed transition [67]. The existence of two conformations may be advantageous in MBP in order to lower the substrate selectivity, and facilitate the recognition of a large variety of maltooligosaccharides, with up seven glucose units [54]. Future studies will be needed to show if the existence of a dynamical equilibrium between multiple stable conformations really provides a functional advantage in hinge-bending proteins. It is also possible that the existence of multiple low energy states facilitate allosteric effects. For example, we found that a displacement of the balancing interface, 20 Å away from the ligand-binding site, had a direct influence on the shape and size of the binding site.

In conclusions, in this paper we presented a combination of conventional and accelerated MD simulations that have been used to gain insight into the pre-existing dynamical equilibrium of conformational states of apo-MBP. The simulations showed that: (1) The previously unobserved semi-closed state is stable on the nanosecond time-scale. Moreover, excellent agreement was obtained between computed PRE rates and experiments, when considering the relative population of the two states at room temperature. (2) Visual inspection of the trajectories and free energy calculations indicated that the balancing interface is displaced during the transformation and acts as a switch that mediates the open- to semi-closed conformational transition. (3) The sharp transition between the open and semi-closed forms is consistent with a model in which the apo protein is in equilibrium between only two, well-defined conformational states. Solvation effects and the packing of non-polar side chains were found to assist the transformation. This interpretation is consistent with a large number of mutational and NMR studies showing an increased affinity for maltose when large non-polar residues are introduced in the hinge region [53], [58].

Because the semi-closed state has a much lower population in apo MBP, it has not been observed previously in X-ray crystal structures. However, the size and shape of the binding site in the semi-closed state appears to be ideally suited for binding to maltodextrins. Therefore, it is possible that the semi-closed conformation, although less populated, is highly relevant for substrate recognition. Ongoing work in our group is focusing on exploring the affinity of both the open and the semi-closed conformation of MBP for different substrates. The simulations presented here provide a structural basis that can be used in future studies to explore the mechanism of substrate recognition in MBP, and in other PBPs. Future studies will reveal if other family members can exchange between open and partially closed conformations, and how to best utilize this phenomenon in drug design protocols.
